# Acellular dermal matrix for rhinophyma: Is it worth it? A new case report and review of literature

**DOI:** 10.1016/j.ijscr.2019.05.013

**Published:** 2019-05-11

**Authors:** Matteo Torresetti, Alessandro Scalise, Giovanni Di Benedetto

**Affiliations:** Clinic of Plastic and Reconstructive Surgery, Department of Experimental and Clinical Medicine, Marche Polytechnic University, Via Conca 71, 60126, Ancona, Italy

**Keywords:** Acellular dermal matrix, Rhinophyma, Dermal substitute, Nasal reconstruction

## Abstract

**Introduction:**

nasal reconstruction after rhinophyma surgery could be challenging. In the last decade, some authors proposed the use of dermal substitutes, but only few case reports have been described throughout the Literature.

**Presentation of case:**

we described a new case of severe and disfiguring rhinophyma treated by total excision and a two-step reconstruction by using acellular dermal matrix and subsequent full-thickness skin graft. Despite an overall improvement of the nasal shape and a good functional recovery were observed after 12 months of follow-up, the aesthetic outcome was not satisfactory and the patient required further surgical revisions.

**Discussion:**

the use of ADMs in rhinophyma poses some important aspects to be discussed. The resorption rate of the matrix and retraction rate of the skin graft make the final thickness of the neodermis unpredictable. Moreover the location of phymatous lesions and the extent of the surgical removal may strongly impact the final aesthetic outcome, often leading to a multistep procedure and patient dissatisfaction.

**Conclusion:**

although we believe that ADM represents a simple and reliable alternative for surgical reconstruction after rhinophyma, basing on our clinical experience we suggested some important tips and tricks in order to avoid surgical revisions, and both surgeons and patients should be aware about the potential drawbacks of this technique.

## Highlights

• Final thickness of the neodermis after ADM application is unpredictable.
• ADM should be restricted to large lesions involving more than one half of the nose.
• For smaller rhinophyma, or lesions of the nasal tip, flap surgery is suggested.
• Excision should be performed as wide as possible on the healthy tissue.
• Lipofilling after ADM reconstruction should be often considered.

## 1 Introduction

Rhinophyma is considered to be the fourth stage of evolving rosacea as described by Rebora [1]. Clinically, the nasal skin is irregularly thickened, with associated erythema and telangiectasia. In severe cases, the skin can have pits, fissures, and scarring with enlarged pores. Tumorous growths can develop in late, nodular forms, producing dramatic cosmetic deformity and occasional functional problems (nasal airway obstruction with obstructive sleep apnea). The lower half of the nose is usually involved, and bony and cartilaginous frameworks are unaffected [2].

Several treatment options have been described in the Literature. Radiotherapy has been largely abandoned owing to its association with secondary skin malignancies [3,4], while medical treatments with antibiotics or retinoids (i.e. isotretinoin to suppress sebum secretion) are useful only in the very early stages. Therefore, there is general agreement that the mainstay of treatment for rhinophyma remains surgical removal of the hypertrophied tissue [2]. Surgical treatment includes total eradication (full thickness excision) or subtotal eradication (partial thickness excision or ‘decortications’) [5]. Subtotal eradication or decortication by tangential excision of diseased tissue has been largely performed by using cryosurgical techniques, dermabrasion, chemical peels, cold scalpel, ultrasonic scalpels, hot wire loops, electrocautery, argon and CO2 laser ablation, radiofrequency blade vapourisation and the Versajet^TM^ Hydrosurgery System [[6], [7], [8]]. Despite this large number of available techniques, total eradication is still considered in case of infiltrating rhinophyma, in the fibrous variant or rhinophyma with underlying skin cancer, and usually requires flap coverage or skin grafts [5]. In the last decade, some authors proposed the use of dermal substitutes for rhinophyma surgery, but only few case reports have been described throughout the Literature [[9], [10], [11]]. We described a new case of severe and disfiguring rhinophyma treated by total excision and acellular dermal matrix (ADM)-based reconstruction, with a 12-months postoperative follow-up. Basing on this clinical experience, the authors proposed some aspects to be discussed in order to avoid surgical complications and to obtain successful results. The present work has been reported in line with the SCARE criteria [10].

## 2 Presentation of case

**Fig. 1:**
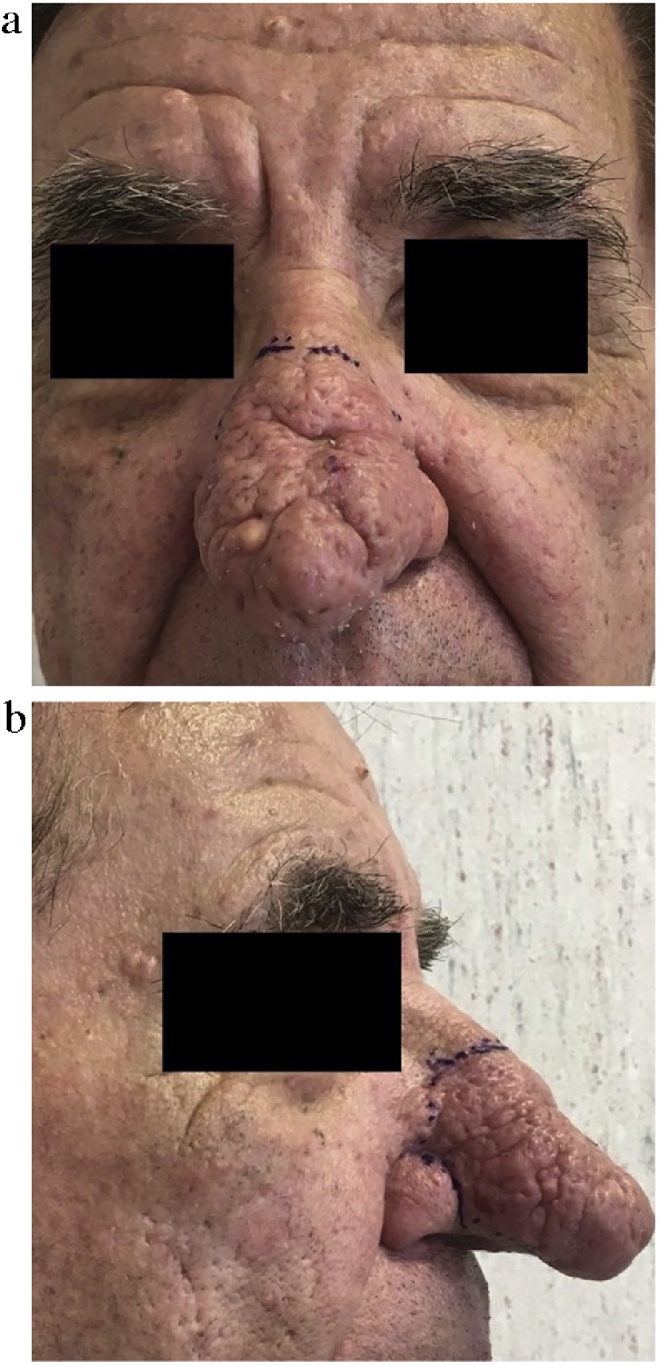
Preoperative anterior (A) and lateral views with marked area of excision (B).

**Fig. 2:**
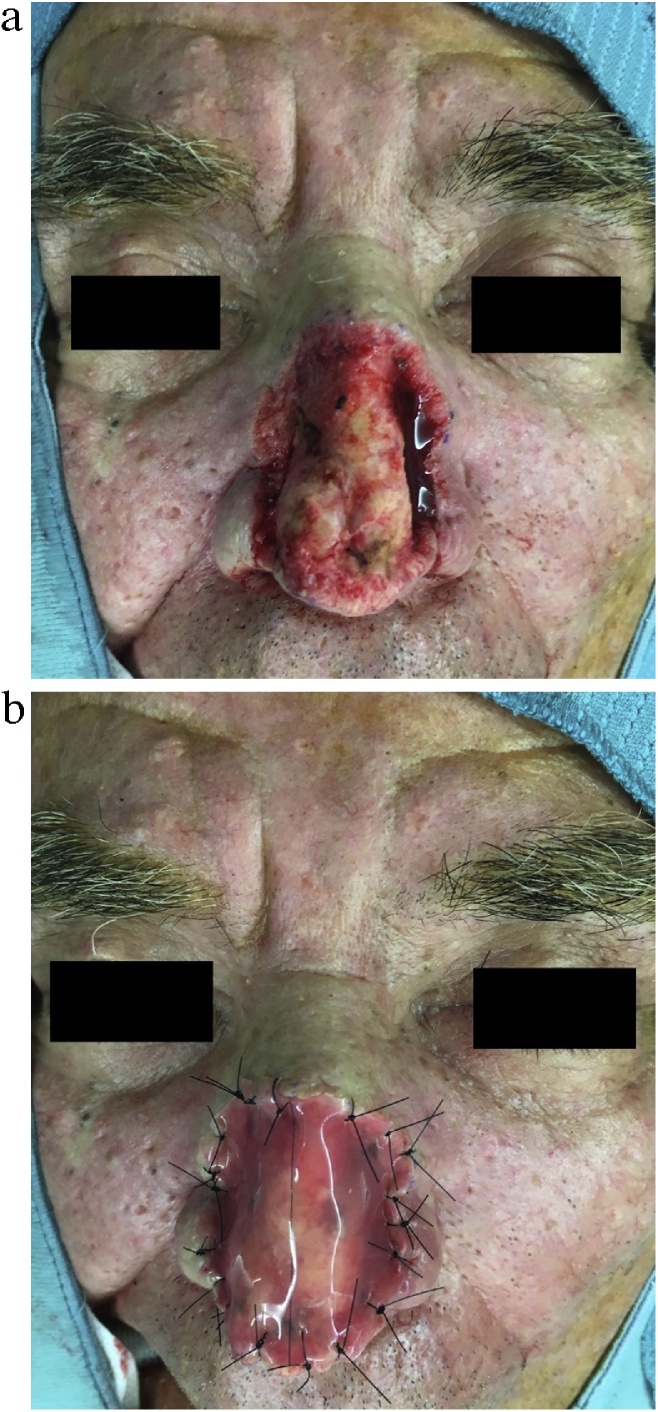
Intraoperative pictures showing the full-thickness excision until the nasal perichondrium (A) and ADM application on the defect area (B).

**Fig. 3:**
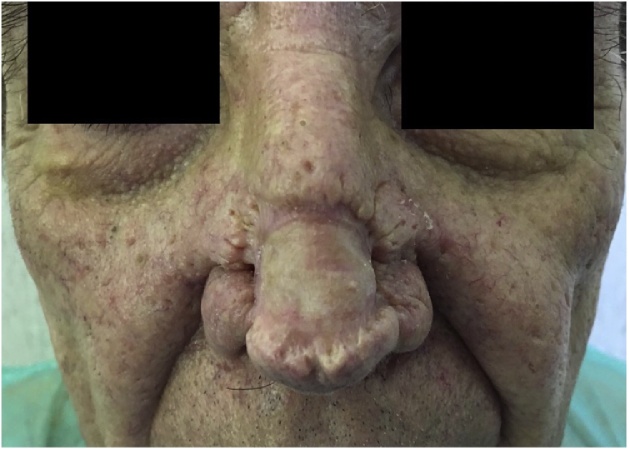
12-months postoperative follow-up.

We present the case of a 77-year-old man with a large tuberous “Cyrano’s like” nose, severe nasal deformity and mild nasal airway obstruction (Fig. 1). It was started about two years before with progressive and irregular thickening of the nasal skin, erythema, and sebaceous secretion. He reported no history of alcohol use, smoke or chronic diseases. Physical examination showed a long tuberous mass mainly involving the lower half of the nose, with complete distortion of the nasal tip and partially the dorsum, and irregular skin surface covered by several pits and grooves. He underwent total surgical excision of the hypertrophied tissue until the nasal perichondrium, and an ADM (Integra^®^ Dermal Regeneration Template, 5 × 5 cm) was placed on the defect area (Fig. 2). Histopathological examination revealed hyperplasia of sebaceous glands, dermal fibrosis with chronic inflammation. The two-step reconstruction was then completed 24 d later with a full-thickness skin graft which completely healed in a few days. The 12-months follow-up visit showed no recurrence of the disease with an overall improvement of the nasal shape and a good functional recovery. Nevertheless, the patient was not satisfied by the aesthetic outcome; therefore he requested further surgical revision due to the different thickness of the reconstructed tissue compared to the surrounding skin with a “step-like effect” (Fig. 3).

## 3 Discussion

Despite surgery offers the only chance of cure by removing the hypertrophied tissue, sometimes nasal reconstruction after extensive removal of diseased tissue may be challenging.

ADM have been largely described in nasal surgery, ranging from resurfacing cutaneous defects following oncologic resection to augmenting the nasal dorsum for aesthetic rhinoplasty [9]. Recently, some authors proposed ADM also for nasal reconstruction after rhinophyma surgery. In 2013 Selig et al described 5 cases of rhinophyma successfully treated by full-thickness excision and 1-step reconstruction by using Matriderm® and split-thickness skin graft [11]. In 2015 Özkan et al reported another case of rhinophyma treated with an electrosurgical device (PlasmaBlade) and single stage reconstruction with Matriderm® and split-thickness skin graft [12]. In 2018 Merigo et al described a single case of laser-assisted excision of phymatous tissue and single-session reconstruction with Matriderm® without skin graft [13]. All these authors reported satisfactory functional and aesthetic outcomes, and no further surgical revisions were necessary.

Basing on these observations, we attempted a new case of rhinhopyma treated by full-thickness excision and 2-step reconstruction with ADM and full-thickness skin graft. To our knowledge, the present case is the first report of Integra®-based reconstruction after rhinophyma surgery, while Matriderm® was used in the previous reports. Although we believe that ADM represents a valid alternative for surgical reconstruction after rhinophyma, our clinical experience suggested some important considerations in order to avoid surgical revisions.

First, although ADM may reduce the retraction rates of skin grafts, final thickness of the neodermis remains unpredictable. In fact it can become much thinner than the immediate postoperative period. A recent systematic review highlighted that one potential drawback to the use of ADM in nasal surgery is the potential for partial resorption of the biologic implant over time. Long-term follow-up showed good results, although partial graft resorption (defined to be up to 50 percent) occurred in 45 percent of patients [9]. Therefore the authors advocated for using ADM sheets that were thicker than 1 mm.

Second, in our opinion ADM-based reconstruction for rhinophyma should be restricted to large lesions involving more than one half of the nose, thus requiring the total reconstruction of the entire aesthetic unit of the nose. For smaller lesions, or rhinophymas confined to the nasal tip, the use of flap surgery with a “like with like” reconstruction is suggested, in order to avoid an unpleasant “patch-like effect”.

Third, excision should be performed as wide as possible on the healthy tissue, thus eliminating the hypertrophied perilesional tissues and minimizing the discrepancy with the reconstructed area. Furthermore, a wider radical excision may reduce the theoretical risk of recurrence with partial excision due to incomplete removal of tissue.

Fourth, lipofilling after ADM reconstruction should be always considered in order to improve the final thickness, elasticity and the overall quality of the reconstructed tissue. Nevertheless, a multistep reconstruction should be performed (at least 3 procedures), with higher costs and lower compliance of the patients.

All these factors may strongly impact the final aesthetic outcome, leading to unespected results and both patient and surgeon dissatisfaction.

## 4 Conclusion

The reliability and simplicity of ADM for nasal reconstruction are now well known; however, their use for rhinophyma should be reserved for selected cases. The authors believe that ADM may be a valuable tool for rhinophyma surgery; nevertheless several aesthetic refinements may be necessary and both surgeons and patients should be aware. Therefore an adequate preoperative evaluation of the lesion locations and a proper surgical planning are mandatory to get optimal patient selection and successful results.

## Conflicts of interest

The authors declare that there is no conflict of interest.

## Funding

The authors received no financial support for the research, authorship, and/or publication of this article.

## Ethical approval

The present study is exempt from ethical approval in our institution.

## Consent

Written informed consent was obtained from the patient for publication of this case report and accompanying images. A copy of the written consent is available for review by the Editor-in-Chief of this journal on request.

## Author contribution

Dr. Matteo Torresetti participated to study design, data collection and interpretation and wrote the paper.

Dr. Alessandro Scalise participated to study design and data collection and interpretation.

Prof. Giovanni Di Benedetto performed the surgical procedure, participated to study design, approved and drafted the final manuscript.

## Registration of research studies

None.

## Guarantor

Prof. Giovanni Di Benedetto.

Dr. Matteo Torresetti.

Dr. Alessandro Scalise.

## Provenance and peer review

Not commissioned, externally peer-reviewed.
